# Liposome-Based Drug Delivery Systems in Cancer Research: An Analysis of Global Landscape Efforts and Achievements

**DOI:** 10.3390/pharmaceutics16030400

**Published:** 2024-03-14

**Authors:** Islam Hamad, Amani A. Harb, Yasser Bustanji

**Affiliations:** 1 Department of Pharmacy, Faculty of Health Sciences, American University of Madaba, Amman 11821, Jordan; 2 Department of Basic Sciences, Faculty of Arts and Sciences, Al-Ahliyya Amman University, Amman 19111, Jordan; 3 Research Institute of Medical and Health Sciences, University of Sharjah, Sharjah 27272, United Arab Emirates; 4 College of Medicine, University of Sharjah, Sharjah 27272, United Arab Emirates; 5 School of Pharmacy, The University of Jordan, Amman 11942, Jordan

**Keywords:** liposomes, drug delivery, cancer, bibliometric, siRNA, gene delivery

## Abstract

Lipid-bilayer-based liposomes are gaining attention in scientific research for their versatile role in drug delivery. With their amphiphilic design, liposomes efficiently encapsulate and deliver drugs to targeted sites, offering controlled release. These artificial structures hold great promise in advancing cancer therapy methodologies. Bibliometric research analyzes systematic literary data statistically. This study used bibliometric indicators to examine, map, and evaluate the applications of liposomes in cancer therapy. A Scopus search was conducted to identify all English-language peer-reviewed scientific publications on the applications of liposomes in cancer therapy within the past twenty years. Bibliometric indicators were calculated using VOSviewer and Biblioshiny. We produced thematic, conceptual, and visualization charts. A total of 14,873 published documents were obtained. The procedure of keyword mapping has effectively identified the main areas of research concentration and prevailing trends within this specific field of study. The significant clusters discovered through theme and hotspot analyses encompassed many topics such as the use of multiple strategies in chemotherapy and different forms of cancer, the study of pharmacokinetics and nanomedicine, as well as the investigation of targeted drug delivery, cytotoxicity, and gene delivery. Liposomes were employed as drug delivery systems so as to selectively target cancer cells and improve the bioavailability of anticancer drugs. The work showcased the capacity to tailor these liposomes for accurate drug delivery by including potent anticancer medications. Our findings not only bring attention to the latest progress in utilizing liposomes for cancer treatment but also underscore the vital need for ongoing research, collaborative efforts, and the effective translation of these breakthroughs into tangible clinical applications, emphasizing the dynamic and evolving nature of cancer therapeutics.

## 1. Introduction

Cancer, a significant and pressing global health concern in the present era, continues to require innovative and creative strategies. Within the perpetual conflict, the domain of nanotechnology has emerged as a promising prospect. Liposomes have become significant assets in the ongoing effort to improve cancer detection and therapy within the field of nanomaterials [1,2,3,4,5]. 

Liposomes, spherical structures made up of one or more layers of phospholipids, are now widely used in drug delivery, especially in cancer treatment [6]. Due to their distinctive composition, they can effectively contain various therapeutic substances, such as both water-soluble and water-insoluble medications, thus improving the solubility and availability of compounds with low water solubility. Liposomes are biocompatible and flexible, so they can be used to deliver medications directly to tumor sites. This makes chemotherapy less harmful as they deliver high concentrations of drugs to the tumor while avoiding healthy tissues [1]. Recent developments in liposome technology have focused on surface modification to avoid recognition by the immune system and prolong circulation, as well as on creating liposomes that respond to specific triggers in the tumor microenvironment to release their contents [7]. This novel method of drug administration showcases the potential of liposomes to enhance the effectiveness of cancer treatments and minimize their adverse effects, representing a notable advancement in the development of more efficient and patient-friendly cancer therapies [8]. These formulations enhance solubility and protect against degradation, while surface changes enable precise delivery to cancer cells, thereby preserving healthy tissues. The enhanced precision of chemotherapy minimizes its harmful consequences, hence enhancing patient outcomes [1]. Moreover, liposomes have the capability to prolong the circulation of drugs, thus enabling sustained release at tumor locations, thereby enhancing the effectiveness of treatment. Moreover, stimuli-responsive designs effectively augment delivery accuracy by enabling the release of medications in response to tumor-specific conditions, hence enhancing the efficacy and patient-friendliness of cancer treatments [6].

Liposome drug delivery systems for cancer treatment encounter many obstacles that impact their practicality in clinical settings. Instability problems, including drug leaking while stored, reduce liposomes’ effectiveness and longevity [8]. It is challenging to achieve selective permeability for targeted drug release without early leakage due to their capacity to encapsulate many medications [1]. Moreover, certain liposome formulations can elicit immunological reactions or toxicity, particularly when used repeatedly [9]. The fabrication process is complicated and expensive, which restricts accessibility. Liposomes’ efficacy in targeting tumors is hindered by the variability of tumor blood vessels and quick removal from the bloodstream by the mononuclear phagocyte system, which reduces drug delivery to the tumor site [10]. Cancer cells can develop resistance mechanisms against medications, which can impact the effectiveness of treatment [11]. Current research is dedicated to the development of drug delivery systems based on liposomes, with a specific emphasis on advancements in composition, targeting mechanisms, and stimuli-responsive designs to boost their effectiveness in cancer therapy.

Functional biopolymers have been recognized as a viable solution to these challenges, resulting in significant progress in the development of biopolymer–liposome hybrids’ promise [12,13]. The new methods include surface-modified liposomes, biopolymer-integrated liposomes, and specialty formulations including liposomes enclosed within hydrogels, films, nanofibers, and pH-sensitive liposomes [14,15,16,17,18]. Polymeric liposomes improve the encapsulation, protection, and targeted release of useful compounds such as vitamins, peptides, and medicines by combining biopolymers with lipid bilayers [19,20,21]. Liposome-based delivery systems have been applied in the biomedical, pharmaceutical, cosmetic, and functional food industries, demonstrating their adaptability and promise [12]. 

Hybrid liposome-based systems combine the natural compatibility and effectiveness of liposomes in encapsulating drugs with various materials like polymers, metallic nanoparticles, and biomolecules. The process of integration has resulted in the development of versatile platforms that greatly improve the administration and effectiveness of cancer treatments. Liposome–polymer hybrids have demonstrated potential in delivering prolonged drug release and enhanced stability, which are essential for improving the therapeutic effectiveness of anticancer drugs [12]. Liposome–metal nanoparticle hybrids possess the ability to perform both therapeutic and diagnostic tasks. This allows for targeted tumor cell therapy and real-time monitoring of treatment effectiveness. The integration of targeting moieties into these hybrids has emerged as a significant advancement, facilitating the accurate administration of drugs to malignant tissues, consequently mitigating unintended consequences and enhancing patient results [22]. One of the noteworthy advancements in the field is the creation of mesoporous silicon microparticles that are incorporated with PEGylated liposomes. This development serves as an illustration of a unique multistage vector strategy for the purpose of targeted treatment of colorectal cancer [23]. Furthermore, the incorporation of functional biopolymers in biopolymer–liposome hybrids effectively overcomes the inherent constraints of traditional liposomes, thereby augmenting the processes of drug encapsulation, protection, and controlled distribution [24]. In addition, the introduction of smart stimuli-responsive liposomal nanohybrid systems represents a notable advancement in cancer theranostics [25]. These systems combine the abundant loading capacity of liposomes with the accuracy of metal nanocarriers to enhance the targeting and effectiveness of therapeutic interventions [25]. The progress made in hybrid liposome technology not only emphasizes its promise in improving cancer treatment tactics, but also demonstrates its usefulness in other diseases, indicating a new era of precise and regulated therapeutic administration [26].

A diverse strategy is required for the management of cancer due to its complexity and heterogeneity. This comprehensive strategy sparked interest in nanotechnology, which gave rise to the discovery of liposomes and lipid-based nanoparticles—nanoscale objects that have extraordinary potential for advancing the diagnosis and treatment of cancer [4,19]. The ability of these lipid-based carriers to encapsulate a variety of medicinal substances, such as chemotherapeutic medicines and nucleic acids, has been exploited [5]. The efficiency of cancer treatments may be considerably increased by being able to deliver these drugs precisely to target areas within the body, such as tumor cells [27,28,29]. Additionally, the fact that these nanoparticles are lipid-based makes them advantageous in terms of the drug solubility, less toxicity, and enhanced bioavailability of anticancer drugs [6,30]. Nevertheless, similar to other domains of innovation, the transition from study to practical implementation is not devoid of obstacles. Limitations have been encountered in traditional techniques to the production and utilization of liposomes in cancer therapy [31]. A number of conventional approaches include complex and resource-intensive procedures, giving rise to issues regarding their efficacy, environmental consequences, and long-term viability [31,32].

Cancer research, particularly in relation to liposomes, is progressing rapidly. In order to stay up to date, it is crucial to perform a comprehensive assessment of the current research, comprehending the connection between novel synthesis techniques, nanoparticle characteristics, and their potential applications in cancer treatment. We employ bibliometric analysis to thoroughly investigate liposome research in cancer, with the objective of defining the present research landscape and emphasizing prospective avenues. This methodology facilitates the identification of prominent research trends, notable contributors, collaborative networks, citation patterns, and the identification of hotspots, thus providing vital insights for researchers, healthcare professionals, and policymakers within this important area [33,34,35,36,37,38,39,40]. 

## 2. Materials and Methods

### 2.1. Search Plan and Refining the Retrieved Documents

On 22 October 2023, we searched the Scopus database to find and evaluate global research outputs related to liposomes and lipid-based NPs in cancer studies throughout the preceding two decades. The following search query (“liposome* OR Lipid based nano*”) AND (“cancer OR anti-cancer OR cytotoxicity OR antiproliferative OR cytotoxic”) covered titles, abstracts, and keywords. Only studies that appeared in peer-reviewed English journals between the years of 2002 and 2022 were taken into account. Materials including press releases, reviews, letters, notes, editorials, conference articles, errata, and other publishing kinds were not included in the search. Also eliminated were book chapters, conference proceedings, and publications in books.

### 2.2. Data Export

In order to make subsequent analysis easier, the documents that were gathered were exported in CSV format. Examining bibliometric data required the use of the Scopus website, as well as Microsoft Office Excel 365 (Microsoft Corporation, Version 2210 Build 16.0.15726.20188, Redmond, WA, USA). This allowed for the collection of information regarding various areas of study, as well as publishing journals. The documents were saved as CSV files so that extra processing could be performed.

### 2.3. Bibliometric Analyses and Visualization

The most recent version of the Visualization of Similarities software, VOSviewer 1.6.18, was used in this study to analyze and map the collaborations, keywords, and citations present in the collected documents. The networks’ partnerships across countries, author–author networking, and author keywords were shown using the VOSviewer mapping approach and cluster analysis [37,41]. Furthermore, the cluster density maps were used to depict all keywords. The Biblioshiny program, which is a component of the Bibliometrix package (version 4.1.4), was employed to carry out supplementary investigations on author keywords [34,36]. By utilizing this software, it became feasible to discern patterns and investigate focal areas by examining the trends in author keywords [36].

To ascertain accuracy, we thoroughly verified the analyses of participating countries and keywords through a manual process. Following this, we integrated analogous terms that were previously identified as distinct entities by employing a thesaurus file, thereby establishing a consistent nomenclature. We have implemented VOSviewer and Biblioshiny software tools to carry out these modifications.

## 3. Results

### 3.1. Analysis of Publications by Year

**Figure 1:**
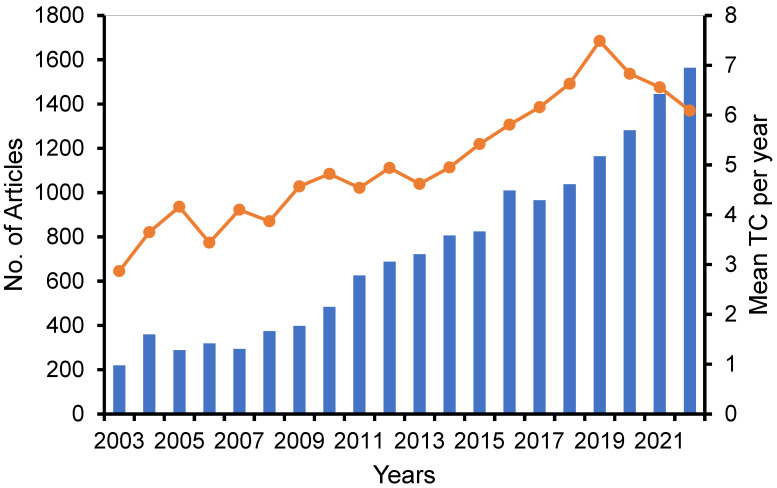
Growth of annual scientific production (lines) and mean TC per year (bar) (total citation divided by number publication divided by years of publication).

A comprehensive search was conducted using the search term (“liposome* OR Lipid based nano*”) AND (“cancer OR anti-cancer OR cytotoxicity OR antiproliferative OR cytotoxic”) to retrieve publications pertaining to the study on the liposomes and solid lipid NPs in cancer therapy from the years 2002 to 2022. This search yielded a total of 14,873 relevant documents. Out of the aforementioned documents, a majority of 6493 (equivalent to 43.7%) were published within the most recent five-year period extending from 2018 to 2022. The curve depicted in Figure 1 illustrates the annual volume of research documents produced.

### 3.2. Analysis of Contributing Journals

A total of 14,873 documents were obtained from a selection of around 1860 Scopus-indexed, peer-reviewed journals. However, only 58 journals have published a minimum of 50 documents. Table 1 presents the rankings of the ten most widely read and actively published journals. Among these, the Journal of Controlled Release stands out as the most productive, having produced 617 publications, accounting for 4.15% of the total. Following closely behind is the International Journal of Pharmaceutics, which has published 531 articles. According to Scopus, the ten highest-ranked journals are categorized as Q1.

### 3.3. Analysis of Articles

The articles that were obtained for analysis exhibit an h-index of 227 and a cumulative citation count of 552,012. On average, each document in the dataset has received 37.1 citations. In addition, it has been observed that a total of 503 documents have received citations amounting to 100 or more. Table 2 displays the top ten documents that have received the greatest number of citations, together with their yearly citation normalization.

### 3.4. Analysis of Authors

The retrieved publications were published with the collaboration of a total of 47,910 authors, with an average of 3.2 contributors per document. Table 3 presents a compilation of the 10 authors who have demonstrated the highest level of activity (data were retrieved directly from Scopus search).

### 3.5. Active Countries

The analysis of our search keywords produced results showing that a total of 95 countries have made contributions to the publication of this specific field within the Scopus database. Table 4 displays the countries that rank highest in terms of publication quantity, including the top 10 nations. There were 20 countries that published 200 papers and above.

The subject has witnessed significant contributions from Chinese academics, who have authored a total of 4205 papers, constituting 28.3% of the overall publications. United States researchers have also made noteworthy contributions, with 3452 documents, representing 23.2% of the total publications. Japanese scholars are ranked third, contributing 1075 articles, which accounts for 7.2% of the overall publications. It is noteworthy to acknowledge that American papers exhibit the largest scientific impact, boasting an average of 55.7 citations per document, thereby surpassing publications from Canada in this regard.

### 3.6. Bibliometric Mapping

#### 3.6.1. International Collaboration

**Figure 2:**
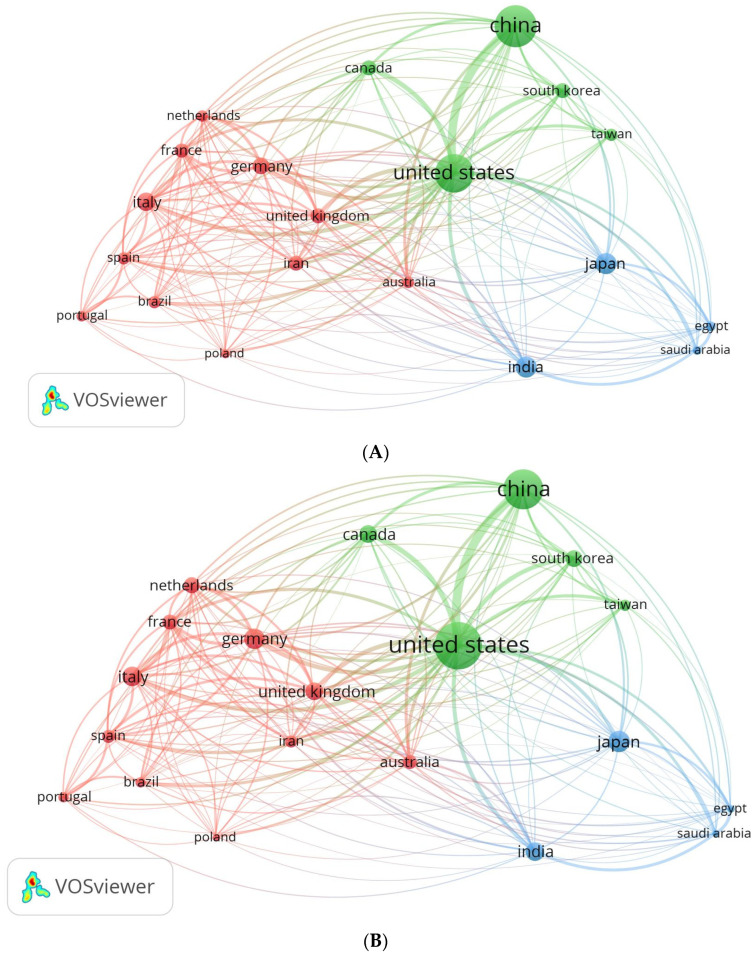
Map of international collaboration as a network visualization. (**A**) Based on the number of documents; (**B**) based on citations.

The VOSviewer software provides valuable insights into the publication and collaboration trends among countries. By utilizing this software, an analysis of international partnerships and collaborations was conducted, resulting in the creation of a network visualization map (Figure 2). The map illustrates countries in the form of spheres, wherein the size of each sphere corresponds to the quantity of published papers (Figure 2A). However, as depicted in Figure 2B, the countries with larger spheres exhibit a greater number of citations on the published publications. Among the 95 countries under consideration, 20 countries managed to meet the requirement of submitting at least 200 publications from their respective nations. The countries were classified into three distinct groups, thereby exemplifying the high level of collaboration among the countries within each respective category. The group consists of eleven countries, namely Germany, the United Kingdom, Iran, the Netherlands, France, Italy, Spain, Brazil, Portugal, Poland, and Australia (highlighted in red). Group 2 comprises five additional countries, namely the United States, China, South Korea, Canada, and Taiwan (shown by the color green). Four countries comprise Group 3 (blue color), including Japan, India, Egypt, and Saudi Arabia. It was observed that authors from the same group of nations frequently choose to publish together, probably because they share a common set of scientific interests. Overall, visualizing international collaborations reveals trends in publication and patterns of international cooperation.

#### 3.6.2. Analysis of Author Keywords and Hotspots Forecasting

To identify active research areas in liposomes application in cancer, we conducted a keyword association analysis using bibliometric tools such as VOSviewer and Biblioshiny. The analysis focused on author keywords, using a thesaurus file to filter out synonyms and a minimum occurrence threshold of 150 times.

**Figure 3:**
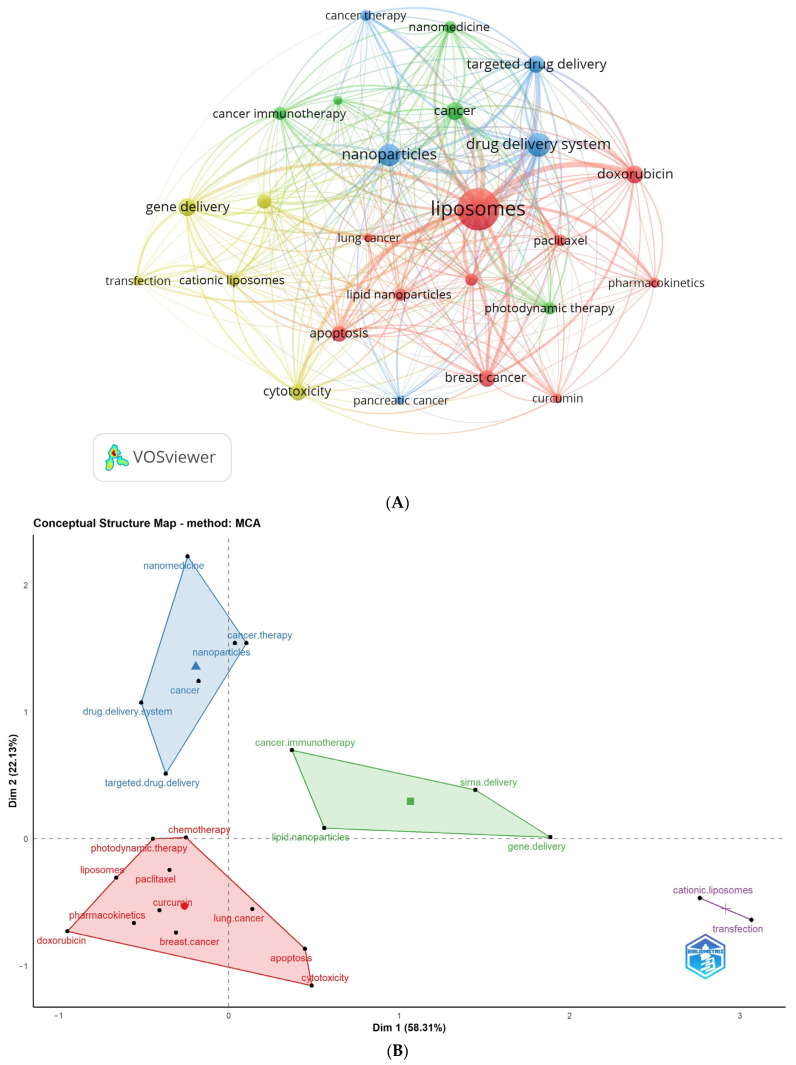
Analysis of the highest occurrence of author keywords. Keywords with a minimum occurrence of 150 times were included: (**A**) Network visualization map obtained by VOSviewer; (**B**) Conceptual structure map obtained by Biblioshiny.

Table 5 and Figure 3 display the most frequent author keywords with a minimum of 150 occurrences. Figure 3A depicts a network visualization map of author keywords, showcasing the most frequent keywords (with 150 or more occurrences) as circular nodes. Nodes of the same color are connected, and their size corresponds to the frequency of connections.

The identified author keywords were organized into four distinct clusters. Cluster 1 (represented in red) includes 10 terms: apoptosis, breast cancer, chemotherapy, curcumin, doxorubicin, lipid nanoparticles, liposomes, lung cancer, paclitaxel, and pharmacokinetics. Cluster 2 (shown in green) comprises keywords related to cancer, cancer immunotherapy, melanoma, nanomedicine, and photodynamic therapy. Cluster 3 (depicted in blue) includes words like cancer therapy, drug delivery system, nanoparticles, pancreatic cancer, and targeted drug delivery. Lastly, Cluster 4 (represented in yellow) encompasses words related to cationic liposomes, cytotoxicity, gene delivery, siRNA delivery, and transfection. These clusters provide insights into the main research areas and topics related to liposome application in cancer therapy, facilitating a better understanding of the prominent themes within the field. It is noteworthy to mention that the outcomes derived from both VOSviewer and Biblioshiny, which are two bibliometric software applications, demonstrated an apparent similarity. The observed similarity indicates that these clusters are indicative of the primary study areas within the domain of liposome application in cancer therapy.

Furthermore, a conceptual structural map was employed to assess the clustering of author keywords, utilizing Biblioshiny as the bibliometric tool (Figure 3B). Notably, these clusters align closely with the previously identified clusters, indicating that the interconnections between keywords significantly influence research focuses within the field.

**Figure 4:**
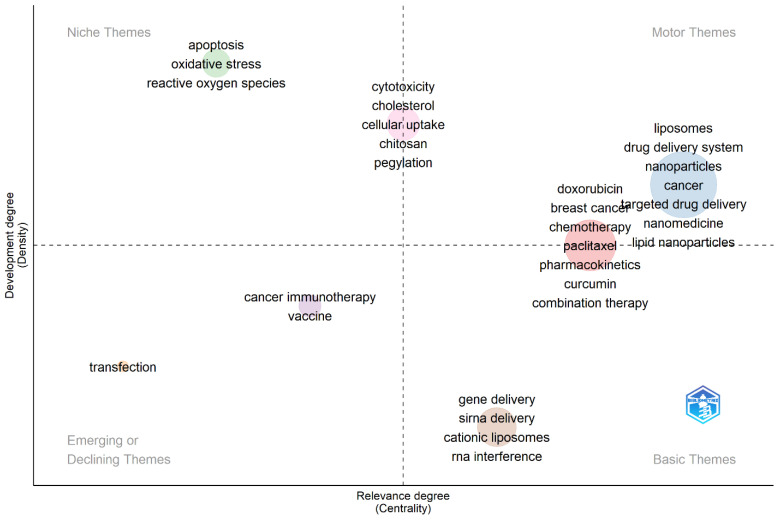
Thematic map acquired from author keyword analyses employing Biblioshiny.

To gain further insights into the extracted keywords, Biblioshiny was utilized to generate a thematic map (Figure 4). Thematic maps are commonly employed in bibliometric studies to explore and present the diverse topics identified within a collection of published materials. The extracted keywords were classified into four overarching thematic groups: niche, motor, emerging, and basic themes. Within the niche themes, apoptosis, oxidative stress, and reactive oxygen species emerged as prominent areas of study. These topics likely represented specialized and focused research within the broader context of liposome application in cancer therapy. The thematic analysis conducted in this study offers valuable insights into the various research themes and topics prevalent in this area of research.

**Figure 5:**
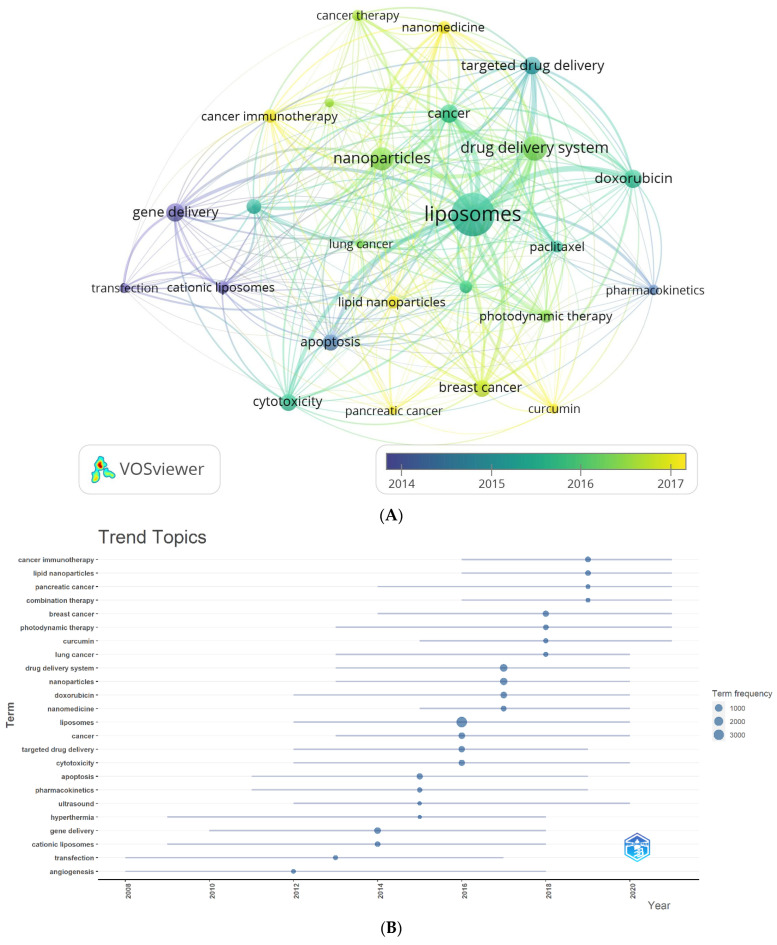

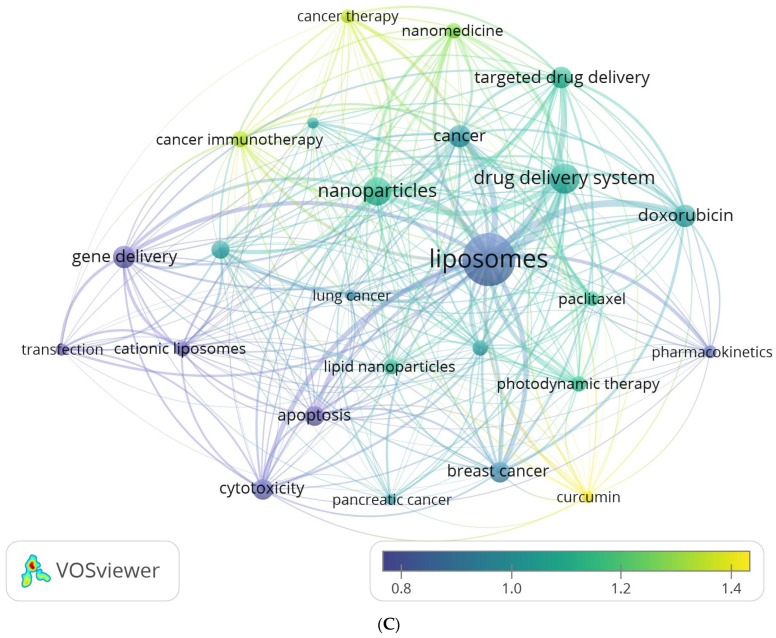
(**A**) Overlay visualization map of the highest occurrence of author keywords with an average publication year overlay. (**B**) Author keyword trends. (**C**) Overlay visualization map of the highest occurrence of author keywords, with an average normalized citations overlay.

Two complementary methods were employed to gain deeper insights into the author’s keyword analysis. The first method involved creating an average publication year-based normalized overlay of keyword clusters, as illustrated in Figure 5A. The color-coded clusters in the figure represent author keywords based on their publication dates, with yellow indicating recently published keywords. This visualization provides a comprehensive overview of how different keyword clusters have evolved. The second method utilized Biblioshiny to visualize trend topics, as shown in Figure 5B. This analysis revealed that cancer immunotherapy, lipid nanoparticles, and pancreatic cancer are the most extensively researched author keyword trends in the last four years of this field. By exploring trend topics, researchers can identify the evolving interests and priorities within liposomes’ application in cancer therapy. Remarkably, both methods yielded consistent findings, reinforcing the robustness of the results. The number of citations varied widely across individual author keywords. To account for the influence of publication age on citation counts, the publication date was used to normalize the citations, ensuring a fair comparison across different periods. Figure 5C displays the normalized mean number of citations for the author keywords under consideration. Among the author keywords, curcumin, cancer immunotherapy, cancer therapy, and nanomedicine demonstrated the highest normalized citation counts, indicating their significant impact and visibility within the research literature. These data provide a comprehensive understanding of the temporal trends, topical foci, and citation impacts of author keywords in the field of liposomes’ application in cancer therapy. These insights contribute to the growing knowledge base and aid researchers in identifying key areas of interest and potential avenues for future research.

#### 3.6.3. Analysis of All Keywords

**Figure 6:**
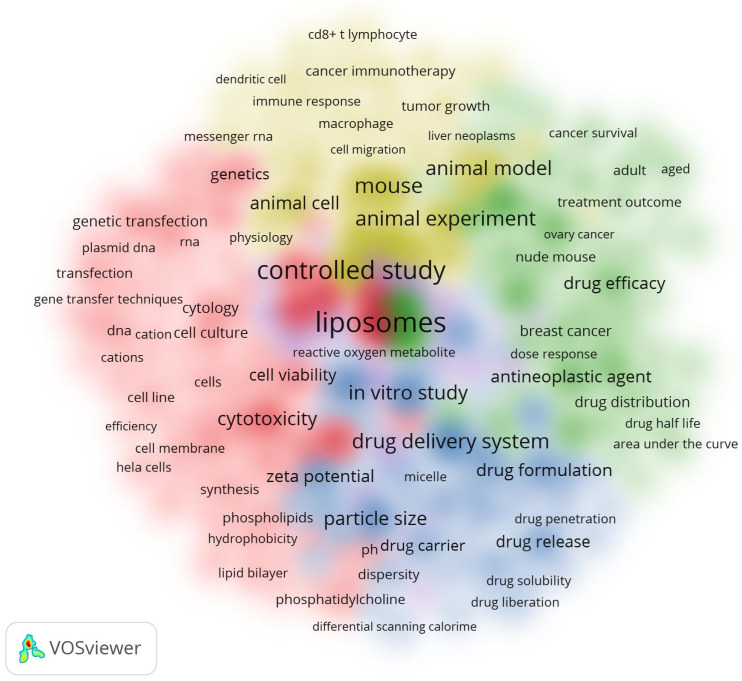
Cluster density of all keywords.

The study also examined the relationships between all keyword terms found in the titles and abstracts of scholarly articles. Figure 6 presents a cluster density map visualization of the co-occurrences of these keywords; a minimum of 300 instances of each keyword was required for inclusion. Among the total of 63,666 words analyzed, only 311 words were found to meet the criteria of having 300 occurrences and more. These words were subsequently visualized in the cluster density map (Figure 6). The relative intensity of the colors on the map corresponds to the frequency of occurrences, whilst words sharing the same color indicate a significant correlation.

## 4. Discussion

Current cancer therapy research has focused on liposomes’ numerous biomedical applications. Nano-scale vesicles are being studied for their possible effects on cancer. Liposomes can be created to deliver targeted drugs using biocompatible compounds [6,32,52,53]. In addition to their ability to be customized, the biocompatibility and aptitude of liposomes to encapsulate many therapeutic compounds make them highly promising options for cancer treatment. The flexibility of liposomes enables the integration of pharmacological agents, hence facilitating the development of innovative combination medicines and personalized treatment approaches [54]. Preliminary developments in liposome cancer therapy demonstrate potential, but further investigation is required to ascertain their complete capabilities and therapeutic viability. Gaining a comprehensive comprehension of liposome-based cancer treatments is of utmost importance in order to enhance treatment modalities and enhance patient outcomes [30,52,53,55,56,57,58].

The analysis of bibliometrics holds significant value in the field of scientific exploration, allowing us to comprehensively examine and visually represent vast bodies of knowledge [38]. In our current research, we aimed to bridge existing gaps in our understanding by offering a quantitative overview of global investigations on liposomes, particularly in the context of cancer therapy research. To achieve this goal, we curated relevant scientific literature from the renowned Scopus database, selected for its reputation as the most extensive repository of peer-reviewed publications, encompassing abstracts and citations. Scopus, with its versatile search options and robust analytical tools, facilitates data extraction for subsequent analysis and visualization. Journals indexed in Scopus undergo stringent peer-review processes and rigorous evaluations, reinforcing their trustworthiness. Furthermore, Scopus classifies these journals based on their subject matter, making it a comprehensive resource for those delving into the realm of liposome applications in cancer therapy research. The results from our study offer valuable perceptions that can guide researchers and institutes in prioritizing their efforts and charting the course for future investigations in this growing field.

This study conducted a comprehensive search to collect all relevant literature pertaining to the research on liposomes’ applications in cancer therapy within the last two decades. This study exclusively incorporated papers and reviews that underwent a rigorous peer-review process and were written in the English language. The findings indicated a notable rise of articles within this particular domain throughout the preceding five-year period, wherein about 43.7% of the papers obtained were published between 2018 and 2022 (Figure 1). A total of 14,873 papers were collected from 1860 journals indexed in Scopus. The top 10 journals, which were the most prolific, accounted for around 20% of the total number of articles disseminated, as shown in Table 1. The documents that were obtained yielded a total of 145,697 citations, with an average of 37.1 citations per document. Additionally, the published publications demonstrated a 227 h-index, which suggests a notable level of reader interest. The Journal of Controlled Release has been shown to possess the maximum number of citations. The documents that have been frequently referenced were released between the time frame of 2004 to 2017, as indicated in Table 2. Nevertheless, the recent publications failed to garner sufficient citations to rival these aforementioned documents. The scholarly publication authored by Olive KP et al., which was published in the journal Science, garnered the most citations out of all the documents that were obtained. The essay examined the delivery of gemcitabine, a chemotherapeutic drug, to pancreatic cancer cells, led to an increase in tumor vasculature, enhanced the delivery of gemcitabine, and caused a delay in disease progression. The aforementioned paper also garnered a high number of citations on a yearly basis.

The examination of the geographic distribution of the retrieved papers reveals that academics from China made the most significant contribution in terms of publications, accounting for 4205 documents (28.3%). Following closely after, researchers from the USA produced 3452 documents (23.2%). The examination of the scientific influence of these nations, as indicated by the average citation per document, indicates that documents originating from the United States exhibit the highest scientific impact (55.7 citation/doc). This is followed by Canada (52.7 citation/document), with Germany and the United Kingdom ranking third with 51.7 citation/doc (Table 4, Figure 2). The retrieved documents garnered a total of 552,012 citations for the purpose of this research. The significance of this topic is evidenced by its substantial citation count, bolstered by the publication of articles in esteemed academic journals. This observation is supported by the data presented in Table 1, which indicate that the top ten publishing journals fall into the Q1 category. International collaborations between nations in this field of study were also examined in the study. The most robust research relationship was found between the USA and China, according to the network visualization of international research collaborations (Figure 2).

**Research trends and hotspots**

VOSviewer and Biblioshiny were used to map hotspots through analyses of the co-occurrences of author keywords in the retrieved literature (more than 150 times). As a result, four overlapping conceptual clusters with 25 main author keywords were identified. These clusters reflect the recovered documents’ areas of interest and focus for their research.

The following hotspots were inferred from the four mentioned clusters obtained from both VOSviewer and Biblioshiny (Figure 4 and Figure 5).

### 4.1. Cluster 1: Liposomal Advancements in Cancer Therapy: Targeting Apoptosis, Breast, and Lung Cancers with Doxorubicin and Paclitaxel, Integrating Curcumin, and Optimizing Pharmacokinetics with Lipid Nanoparticles

Liposomes target apoptosis in cancer cells, making them attractive cancer treatments [59]. Customized lipid vesicles can transport therapeutic chemicals directly to cancer cells, causing apoptosis and limiting tumor growth [60]. Liposomes may improve cancer treatments by targeting programmed cell death, reducing negative effects on healthy organs. In the battle against breast and lung cancers, liposomes stand out as innovative carriers for chemotherapy drugs like doxorubicin and paclitaxel [61,62]. These lipid spheres serve as specialized delivery vehicles, transporting these potent drugs directly to the cancer cells while minimizing exposure to healthy tissues [18,19]. This targeted approach not only enhances the effectiveness of the chemotherapy but also has the potential to reduce the adverse side effects commonly associated with these powerful medications. Incorporating the natural compound curcumin into liposomes for cancer treatment is a promising frontier, as liposomes offer an ingenious approach to its integration [63]. These lipid carriers provide a tailored delivery system for curcumin, ensuring its targeted arrival at cancer cells. Known for its anti-inflammatory and anticancer properties, curcumin’s potential is maximized when encapsulated within liposomes, enhancing its bioavailability and therapeutic impact [64]. This strategic combination of liposomes and curcumin represents a noteworthy avenue in the ongoing pursuit of innovative and effective cancer therapies. 

Doxorubicin and paclitaxel are widely used chemotherapy drugs for treating different types of cancer, representing major progress in the field of oncology [17,61]. Integrating them into nanoliposome carriers is a promising approach to improve their effectiveness and lower systemic toxicity [53,55]. Nanoliposomes provide targeted drug delivery due to their small size and biocompatibility, allowing a larger concentration of the drug to reach the tumor site while reducing the exposure of healthy tissues [5]. This focused strategy enhances the therapeutic effectiveness against cancer cells while reducing the typical unpleasant side effects of chemotherapy. Moreover, enclosing doxorubicin and paclitaxel in nanoliposomes has been demonstrated to counteract certain drug resistance processes in cancer cells, leading to enhanced treatment results [24,65]. 

Liposomes containing paclitaxel and immunotherapy are crucial in boosting the immune system’s capacity to identify and eliminate cancer cells in cancer immunotherapy [66]. Precise medication delivery can alter the tumor microenvironment to enhance its vulnerability to immune cell assault [66]. Furthermore, these drugs, doxorubicin and paclitaxel, can be formulated combined with other chemical compounds to release their contents under a certain microenvironment, enabling accurate regulation of medication release and reducing harm to nearby healthy tissues [67]. This combined treatment approach utilizing chemotherapy, immunotherapy, and photodynamic therapy with the assistance of nanoliposome technology presents a comprehensive strategy for cancer treatment, with the possibility of enhancing effectiveness and patient results [68,69].

Recent studies have examined the synergistic benefits of mixing conventional chemotherapeutic drugs such as doxorubicin or paclitaxel with natural chemicals to improve their effectiveness and minimize the adverse effects of cancer therapies. The research highlights how natural substances might enhance the effectiveness of traditional chemotherapy as adjuvants, leading to improved treatment results. The potential of combining antitumoral medicines with various natural chemicals for cancer treatment has been studied, emphasizing the importance of doxorubicin or paclitaxel co-treatment regimens [70,71]. These combinations are created to make use of the distinct characteristics of each component, with the goal of affecting different pathways related to cancer advancement. This approach aims to enhance the effectiveness of treatment while reducing potential harm.

Researchers hope to enhance the inhibition of cancer cell development by combining natural chemicals like curcumin, quercetin, and resveratrol with effective chemotherapeutic drugs in nanoliposomes to capitalize on their synergistic effects [72,73]. These natural chemicals possess anti-inflammatory, antioxidant, and anti-cancer characteristics that, when used in conjunction with doxorubicin and paclitaxel, can enhance their therapeutic results [74,75,76]. Curcumin has been demonstrated to increase the responsiveness of cancer cells to chemotherapy, making it a beneficial component in liposome formulations [71]. This novel approach has been utilized in the management of several forms of cancer, such as breast, ovarian, and lung malignancies, providing a promising solution for patients in search of more efficient and less harmful treatment alternatives [64]. These liposome formulations have potential applications beyond cancer therapy, demonstrating the adaptability of combining standard chemotherapeutic drugs with natural chemicals in sophisticated drug delivery systems for treating many disorders.

The exploration of pharmacokinetics with lipid nanoparticles introduces a sophisticated dimension to drug delivery optimization. Pharmacokinetics, encompassing the absorption, distribution, metabolism, and excretion of drugs within the body, is finely tuned with the integration of lipid nanoparticles [53,77]. Serving as adept carriers, these nanoparticles enhance drug solubility and stability, addressing critical challenges in pharmaceutical formulations. Moreover, their capacity to efficiently navigate physiological barriers augments drug absorption and distribution profiles. This strategic synergy not only refines drug formulations but also holds promise in optimizing drug concentrations at target sites, thereby advancing therapeutic outcomes [78].

### 4.2. Cluster 2: Liposomal Advancements: Propelling Cancer Immunotherapy, Nanomedicine Precision, and Photodynamic Therapy across Oncology

Lipid nanoparticles are considered a complex approach to cancer immunotherapy. These microscopic lipid spheres act as effective carriers, transporting therapeutic agents precisely to the immune system’s attention [79]. Liposomes serve as vehicles for delivering immune-stimulating substances, arranging a targeted and potent response against cancer cells [80]. By encapsulating immunomodulatory agents, liposomes enhance the therapeutic payload, maximizing the body’s ability to recognize and combat cancer. This strategic alliance between liposomes and cancer immunotherapy represents a promising frontier, elevating the precision and efficacy of treatments in the ongoing quest to conquer cancer [81]. Liposomes can be designed to encapsulate a combination of pembrolizumab, an immune checkpoint inhibitors, and pegylated liposomal doxorubicin [82]. This allows for tailored delivery to the tumor microenvironment, enhancing the effectiveness of these inhibitors while minimizing side effects. This blend utilizes nanotechnology’s precision along with immunotherapy’s capacity to more effectively impair cancer’s ability to avoid the immune system. Liposome-encapsulated ICIs have demonstrated potential in treating several types of cancer such as ovarian cancer, melanoma, non-small cell lung cancer, and renal cell carcinoma, indicating a wide range of effectiveness across different cancer types [83,84]. This novel approach enhances the therapeutic effectiveness of ICIs and allows for combining them with other anticancer agents in liposomes to achieve a synergistic effect, potentially improving treatment outcomes and patient survival rates.

Liposomes in photodynamic therapy (PDT) are an innovative method in oncology that provides a precise and less intrusive treatment alternative for different types of malignancies [67]. PDT utilizes photosensitizing chemicals that, when activated by particular light wavelengths, generate reactive oxygen species that can eliminate cancer cells, harm blood arteries within the tumor, and stimulate the immune system. Liposomes are an optimal method for delivering these photosensitizers, improving their solubility, stability, and targeting ability while decreasing their overall toxicity in the body [85]. Numerous photosensitizing agents have been documented in cancer research, including photofrin, aminolevulinic acid (ALA) and its derivatives, temoporfin, chlorin e6 (Ce6), phthalocyanines, and naphthalocyanines [67,86,87,88].

Liposomal formulations of photosensitizers have been designed to treat skin cancers including basal cell carcinoma. When applied topically, these formulations enhance medication penetration and retention at the tumor site [86]. Furthermore, liposomal photosensitizers can be given intravenously for advanced tumors such as lung and esophageal cancer [89]. The liposomes gather in the tumor tissue because of the increased permeability and retention effect. This targeted delivery method enables the specific activation of the photosensitizer through exposure to light, reducing harm to nearby healthy tissues [30,67,85]. Moreover, due to the adaptability of liposomes, multifunctional nanocarriers capable of transporting photosensitizers and chemotherapeutic drugs concurrently have been made possible, resulting in a synergistic therapeutic effect. The prospective treatment outcomes for diverse oncological landscapes are suggested by the results observed in preclinical models of this combination therapy approach. Liposomal PDT has the potential to be an effective and minimally invasive treatment option for cancer patients, as evidenced by the ongoing research and development in this field [30,67,85].

### 4.3. Cluster 3: Enhancing Targeted Cancer Therapy: The Role of Liposomes in Precision Drug Delivery

In drug targeting, liposomes have emerged as advanced tools in the domain of targeted drug delivery [90]. These lipid vesicles exhibit a unique capacity to encapsulate a diverse array of therapeutic agents, facilitating their transport to specific cells or tissues with enhanced precision [91]. For instance, in cancer therapy, liposomes can encapsulate chemotherapeutic drugs, allowing for targeted release at tumor sites while minimizing systemic exposure and associated side effects [79]. The phospholipid bilayers of liposomes can be tailored for controlled drug release, optimizing therapeutic efficacy. This strategic alliance between liposomes and targeted drug delivery is exemplified by real-world applications, such as the use of liposomal doxorubicin formulations in oncology, underscoring their potential to revolutionize drug delivery paradigms for improved patient outcomes [55,61,92]. Understanding the complexities of pancreatic cancer, a powerful opponent in the field of oncology, requires the use of novel tactics. Liposomes offer a promising solution in this difficult situation [27]. Pancreatic cancer, often diagnosed at an advanced stage, poses unique hurdles due to its location and aggressive nature. Traditional treatments face limitations in reaching the pancreas effectively, but liposomes, with their precision and adaptability, introduce a transformative element [44]. These lipid-based carriers, when loaded with therapeutic agents, hold the potential to breach the barriers presented by pancreatic cancer’s formidable defenses. Whether encapsulating chemotherapy drugs for targeted release or facilitating the delivery of molecular therapies, liposomes address the critical need for site-specific treatments [93]. As researchers investigate the complexities of pancreatic cancer, the strategic integration of liposomes emerges as a pivotal avenue, promising enhanced precision and efficacy in therapeutic interventions, a significant stride towards improving the prognosis for patients grappling with this challenging disease [94].

Liposomes, as drug carriers, have improved the therapy of several types of cancer such as lung cancer, breast cancer, and melanoma, by improving the effectiveness and decreasing the harmful effects of chemotherapy drugs [32,66]. Liposomal formulations of medications like doxorubicin have demonstrated potential in lung cancer by targeting tumor cells more efficiently, reducing harm to healthy lung tissue, and enhancing patient results. Liposomes’ targeted delivery technique enables increased medication concentrations to gather at the tumor site, essential for treating cancers resistant to standard chemotherapy [95]. 

Liposomal encapsulation of chemotherapeutic drugs and immuno-therapeutics have significantly advanced treatment in breast cancer and melanoma [96]. Liposomal anthracyclines have been created for breast cancer to decrease heart toxicity while still being effective against tumors [97]. Liposomes are being investigated for delivering nucleic acid-based treatments and checkpoint inhibitors in cancer to enhance the immune system’s ability to target cancer cells effectively [5,7,98]. An important feature of liposomes is their ability to adapt encapsulating various medications, both hydrophilic and hydrophobic, which makes them a great asset in the battle against cancer [32]. They provide a foundation for combining therapies and developing innovative therapeutic approaches.

### 4.4. Cluster 4: Liposomal progresses: Unraveling the Potential in Cationic Liposomes for Enhanced Gene and siRNA Delivery with Minimized Cytotoxicity and Optimal Transfection Efficiency

Cationic liposomes possess a positive charge, allowing them to interact with negatively charged cellular components, a characteristic advantageous in the context of cancer cells [99]. Cationic liposomes excel in delivering therapeutic payloads, including genes or small interfering RNA (siRNA), precisely to cancer cells, enabling targeted interventions [100]. Beyond their delivery prowess, cationic liposomes display a unique ability to minimize cytotoxicity, a critical consideration in cancer treatment where preserving healthy tissues is paramount [29,101,102]. This strategic combination positions cationic liposomes as promising tools in the ongoing quest for effective and selective cancer therapies. Liposomes are flexible agents in the targeted administration of small interfering RNA (siRNA) in the dynamic field of molecular medicine [19,100]. These lipid spheres offer a protective shield for siRNA molecules, preventing their degradation in the bloodstream and facilitating their precise delivery to specific cells or tissues [103]. In the context of gene silencing and therapeutic interventions, liposomes serve as effective vehicles to navigate biological barriers, enhancing the bioavailability and efficacy of siRNA [100]. This strategic alliance allows researchers and clinicians to fine-tune gene expression with a level of precision previously elusive in traditional therapeutics. The use of liposomes in siRNA delivery represents a promising frontier, holding the potential to unlock new possibilities in the treatment of genetic disorders, infectious diseases, and various cancers, offering a glimpse into the future of personalized and targeted medicine [5]. Liposomes also emerge as invaluable tools for optimizing transfection efficiency. These vesicles excel in transmitting genetic material into target cells, facilitating the delivery of nucleic acids such as DNA or RNA [98,102]. The efficiency of this process, known as transfection, is vital in manipulating cellular functions and studying gene expression [104]. Liposomes, with their ability to encapsulate and protect genetic material, enhance transfection efficiency by ensuring the successful integration of foreign genetic material into the host cells. This strategic use of liposomes not only streamlines experimental procedures in laboratories but also holds profound implications for the development of gene therapies and advancements in understanding cellular mechanisms [105]. The collaboration between liposomes and transfection efficiency represents a pivotal aspect in molecular research, propelling the boundaries of what is achievable in the manipulation and understanding of genetic information within living cells [78]. Recent studies have highlighted the considerable impact of liposome-based delivery systems on the advancement of cancer immunotherapy, specifically in the context of chimeric antigen receptor (CAR) T-cell treatment [106]. CAR T-cell therapy is a significant breakthrough in the field of cancer immunotherapy, providing a new and powerful method for treating many forms of cancer, with a special focus on hematologic malignancies [106]. This treatment approach entails the genetic alteration of a patient’s T-cells to produce a CAR that selectively targets cancer cells. Numerous studies have demonstrated that the utilization of liposomes for the encapsulation of adenovirus yields substantial improvements in the transport and efficacy of CAR T-cell treatment. This, in turn, results in noteworthy reductions in tumor size and heightened levels of anti-cancer immunity. Subsequent research on lipid nanovesicle systems demonstrates their capacity to specifically target tumors with immunotherapy medicines, hence enhancing the effectiveness of CAR-T therapy [106,107,108]. 

## 5. Study Limitations

Since our study solely used Scopus to extract the relevant documents, it is important to note that significant research papers on liposomes’ applications in cancer therapy may have been published in publications that were not included in Scopus. Furthermore, a limited number of papers from 2022 were possibly not yet included in Scopus at the time of our research, and it is probable that they were left out of our analysis. Furthermore, our search did not include items that were still undergoing the “in press” phase. Therefore, it is likely that certain research articles or review papers in the subject, released in late 2022, may have been disregarded as a result of these conditions. Another drawback of our analysis is that we solely included papers that were published in the English language. Research undertaken in different languages could provide researchers with valuable insights into the applications of liposomes in cancer therapy. Finally, it is important to acknowledge that even small inaccuracies in the authors’ names or affiliations could have an impact on the results of our study.

## 6. Conclusions and Future Directions

In conclusion, this comprehensive bibliometric analysis sheds light on the remarkable potential of liposomes’ applications in cancer therapy in various scientific domains. Our exploration into the current state of research and future directions underscores the transformative impact of liposomes. The potential of liposomes and lipid nanoparticles in detecting and treating cancer has emerged as a key focus, offering the tantalizing prospect of enhanced patient outcomes with reduced adverse effects. Furthermore, charged liposomes may allow the interaction with charged cellular components, a characteristic advantageous in the context of cancer cells.

In essence, our research emphasizes the importance of further study, cooperation, and practical implementation of liposomal applications in oncology due to their developments and potential. This bibliometric analysis outlines the current status of liposome research in cancer therapy and highlights its significant influence on future therapeutic approaches, emphasizing the need for ongoing progress and collaborative interdisciplinary work to apply research findings. This study demonstrates the significant impact of liposomes in enhancing cancer treatment, representing progress in the search for more efficient and safer healthcare options.

The future of liposome research in cancer therapy is to address current obstacles and maximize their whole therapeutic capabilities. Significant progress has been made in strengthening liposome stability, enhancing targeting accuracy, and creating stimuli-responsive liposomes for precise drug delivery. Incorporating functional biopolymers into liposome structures is a promising approach to enhance drug delivery mechanisms. Studying the combined effects of liposomes and natural chemicals offers a new method to enhance treatment effectiveness and minimize adverse effects in different forms of cancer. Efforts are concentrated on customizing liposome formulations for certain tumors such breast, lung, and melanoma to enhance therapy results. Future research will focus on creating advanced liposome-based treatments that can specifically target cancer cells, reduce harmful side effects, and improve the delivery of drugs. Collaboration across several disciplines and the application of research discoveries into clinical settings are essential for the progress of liposome technology in oncology, offering more effective and patient-focused cancer therapies.

## Figures and Tables

**Table 1:** Top 10 active journals publishing documents.

Source	No of Publication	%	Citations	Scopus Percentile (Q)
Journal of controlled release	617	4.15	39,071	98 (Q1)
International journal of pharmaceutics	531	3.57	20,496	93 (Q1)
International journal of nanomedicine	387	2.60	12,300	96 (Q1)
Biomaterials	341	2.29	27,800	98 (Q1)
Colloids and surfaces B: biointerfaces	241	1.62	7532	90 (Q1)
Molecular pharmaceutics	239	1.61	9832	86 (Q1)
Pharmaceutics	234	1.57	2940	76 (Q1)
Plos ONE	167	1.12	5417	87 (Q1)
Pharmaceutical research	161	1.08	5560	81 (Q1)
Drug delivery	160	1.08	4546	85 (Q1)

**Table 2:** Top ten cited documents on liposome application in cancer therapy.

Paper	Total Citations	TC per Year	Normalized TC
OLIVE KP, 2009, SCIENCE ([42])	2516	167.73	39.31
CABRAL H, 2011, NAT NANOTECHNOL ([43])	1965	151.15	36.08
KAMERKAR S, 2017, NATURE ([44])	1507	215.29	40.74
DING J, 2016, NATURE ([45])	1503	187.88	36.92
ARRUEBO M, 2007, NANO TODAY([46])	1408	82.82	21.48
O’BRIEN MER, 2004, ANN ONCOL ([47])	1357	67.85	19.57
ITO A, 2005, J BIOSCI BIOENG ([48])	1331	70.05	17.79
VAN DER POL E, 2012, PHARMACOL REV ([49])	1301	108.42	23.94
ALKILANY AM, 2010, J NANOPART RES ([50])	1219	87.07	19.43
SUN D, 2010, MOL THER ([51])	1167	83.36	18.61

**Table 3:** Top 10 most productive authors and co-cited authors in liposome application in cancer therapy.

Rank	Author	Documents	Total Citation for Author
1st	Jaafari, M.R.	92	10,717
2nd	Harashima, H.	90	24,792
3rd	Lee, R.J.	81	19,053
4th	Torchilin, V.P.	76	61,532
5th	Zhang, Z.	63	19,235
6th	Zhang, Q.	62	21,758
7th	Storm, G.	59	40,212
8th	He, Q.	54	9270
9th	Oku, N.	45	12,583
10th	Lopez-Berestein, G.	44	36,331

**Table 4:** Top ten active countries in publishing documents on liposome application in cancer therapy.

Rank	Country	Number of Publications	Total Citations	% of Total Documents	Citation/Document
1st	China	4205	136,047	28.3	32.4
2nd	United States	3452	192,391	23.2	55.7
3rd	Japan	1075	40,995	7.2	38.1
4th	India	1030	30,699	6.9	29.8
5th	Italy	779	31,782	5.2	40.8
6th	Germany	697	36,029	4.7	51.7
7th	South Korea	559	23,652	3.8	42.3
8th	United Kingdom	537	27,788	3.6	51.7
9th	Iran	517	12,301	3.5	23.8
10th	Canada	517	27,227	3.5	52.7

**Table 5:** Highly frequent keywords (150 times and more).

Author Keyword	Frequency	Cluster
apoptosis	457	1
breast cancer	485	1
chemotherapy	272	1
curcumin	181	1
doxorubicin	591	1
lipid nanoparticles	282	1
liposomes	3305	1
lung cancer	162	1
paclitaxel	269	1
pharmacokinetics	200	1
cancer	593	2
cancer immunotherapy	319	2
melanoma	151	2
nanomedicine	299	2
photodynamic therapy	282	2
cancer therapy	220	3
drug delivery system	1029	3
nanoparticles	917	3
pancreatic cancer	151	3
targeted drug delivery	553	3
cationic liposomes	282	4
cytotoxicity	498	4
gene delivery	576	4
siRNA delivery	399	4
transfection	182	4

## Data Availability

All data generated or analyzed during this study are encompassed in the submitted article.

## References

[1] Nsairat H., Khater D., Sayed U., Odeh F., Al Bawab A., Alshaer W. (2022). Liposomes: Structure, composition, types, and clinical applications. Heliyon.

[2] Alshaer W., Zraikat M., Amer A., Nsairat H., Lafi Z., Alqudah D.A., Al Qadi E., Alsheleh T., Odeh F., Alkaraki A. (2019). Encapsulation of echinomycin in cyclodextrin inclusion complexes into liposomes: In vitro anti-proliferative and anti-invasive activity in glioblastoma. RSC Adv..

[3] Matalqah S.M., Aiedeh K., Mhaidat N.M., Alzoubi K.H., Bustanji Y., Hamad I. (2020). Chitosan nanoparticles as a novel drug delivery system: A review article. Curr. Drug Targets.

[4] Fernandes D.A. (2023). Liposomes for Cancer Theranostics. Pharmaceutics.

[5] Gao Y., Liu X., Chen N., Yang X., Tang F. (2023). Recent Advance of Liposome Nanoparticles for Nucleic Acid Therapy. Pharmaceutics.

[6] Nikolova M.P., Kumar E.M., Chavali M.S. (2022). Updates on Responsive Drug Delivery Based on Liposome Vehicles for Cancer Treatment. Pharmaceutics.

[7] Gu Z., Da Silva C.G., van der Maaden K., Ossendorp F., Cruz L.J. (2020). Liposome-based drug delivery systems in cancer immunotherapy. Pharmaceutics.

[8] Chen J., Hu S., Sun M., Shi J., Zhang H., Yu H., Yang Z. (2024). Recent advances and clinical translation of liposomal delivery systems in cancer therapy. Eur. J. Pharm. Sci..

[9] Moghimi S.M., Hamad I. (2008). Liposome-mediated triggering of complement cascade. J. Liposome Res..

[10] Zhang L., Xie G., Xiao X., Cheng C. (2023). Characterization of PDL1 enhanced siRNA/albumin liposome for effective therapeutic function in lung cancer. J. Cancer Res. Clin. Oncol..

[11] Zhu W., Yu H., Jia M., Lin C., Yuan Z., Tan X., Yan P. (2023). Multi-targeting liposomal codelivery of cisplatin and rapamycin inhibits pancreatic cancer growth and metastasis through stromal modulation. Int. J. Pharm..

[12] Tan C., Wang J., Sun B. (2021). Biopolymer-liposome hybrid systems for controlled delivery of bioactive compounds: Recent advances. Biotechnol. Adv..

[13] Bishani A., Makarova D.M., Shmendel E.V., Maslov M.A., Sen’kova A.V., Savin I.A., Gladkikh D.V., Zenkova M.A., Chernolovskaya E.L. (2023). Influence of the Composition of Cationic Liposomes on the Performance of Cargo Immunostimulatory RNA. Pharmaceutics.

[14] Duché G., Heu C., Thordarson P. (2023). Development and Characterization of Nanoscale Gel-Core Liposomes Using a Short Self-Assembled Peptide Hydrogel: Implications for Drug Delivery. ACS Appl. Nano Mater..

[15] Wan H., Wang S., Li C., Zeng B., Wu H., Liu C., Chen L., Jin M., Huang W., Zang Y. (2023). LA67 Liposome-Loaded Thermo-Sensitive Hydrogel with Active Targeting for Efficient Treatment of Keloid via Peritumoral Injection. Pharmaceutics.

[16] Tang J.S.J., Smaczniak A.D., Tepper L., Rosencrantz S., Aleksanyan M., Dähne L., Rosencrantz R.R. (2022). Glycopolymer Based LbL Multilayer Thin Films with Embedded Liposomes. Macromol. Biosci..

[17] Hasanbegloo K., Banihashem S., Faraji Dizaji B., Bybordi S., Farrokh-Eslamlou N., Abadi P.G.S., Jazi F.S., Irani M. (2023). Paclitaxel-loaded liposome-incorporated chitosan (core)/poly(ε-caprolactone)/chitosan (shell) nanofibers for the treatment of breast cancer. Int. J. Biol. Macromol..

[18] Lafi Z., Alshaer W., Hatmal M.M., Zihlif M., Alqudah D.A., Nsairat H., Azzam H., Aburjai T., Bustanji Y., Awidi A. (2021). Aptamer-functionalized pH-sensitive liposomes for a selective delivery of echinomycin into cancer cells. RSC Adv..

[19] Gharaibeh L., Alshaer W., Wehaibi S., Al Buqain R., Alqudah D.A., Al-Kadash A., Al-Azzawi H., Awidi A., Bustanji Y. (2021). Fabrication of aptamer-guided siRNA loaded lipopolyplexes for gene silencing of notch 1 in MDA-mb-231 triple negative breast cancer cell line. J. Drug Deliv. Sci. Technol..

[20] Caritá A.C., Resende de Azevedo J., Chevalier Y., Arquier D., Buri M.V., Riske K.A., Ricci Leonardi G., Bolzinger M.A. (2023). Elastic cationic liposomes for vitamin C delivery: Development, characterization and skin absorption study. Int. J. Pharm..

[21] Dzhumashev D., Anton-Joseph S., Morel V.J., Timpanaro A., Bordon G., Piccand C., Aleandri S., Luciani P., Rössler J., Bernasconi M. (2024). Rapid liposomal formulation for nucleolin targeting to rhabdomyosarcoma cells. Eur. J. Pharm. Biopharm..

[22] Sun L., Fan M., Huang D., Li B., Xu R., Gao F., Chen Y. (2021). Clodronate-loaded liposomal and fibroblast-derived exosomal hybrid system for enhanced drug delivery to pulmonary fibrosis. Biomaterials.

[23] Cevenini A., Celia C., Orrù S., Sarnataro D., Raia M., Mollo V., Locatelli M., Imperlini E., Peluso N., Peltrini R. (2020). Liposome-embedding silicon microparticle for oxaliplatin delivery in tumor chemotherapy. Pharmaceutics.

[24] Pardhi E., Yadav R., Chaurasiya A., Madan J., Guru S.K., Singh S.B., Mehra N.K. (2023). Multifunctional targetable liposomal drug delivery system in the management of leukemia: Potential, opportunities, and emerging strategies. Life Sci..

[25] Alwattar J.K., Mneimneh A.T., Abla K.K., Mehanna M.M., Allam A.N. (2021). Smart stimuli-responsive liposomal nanohybrid systems: A critical review of theranostic behavior in cancer. Pharmaceutics.

[26] Ning S., Zhang X., Suo M., Lyu M., Pan Y., Jiang Y., Yang H., Yip Lam J.W., Zhang T., Pan L. (2023). Platelet-derived exosomes hybrid liposomes facilitate uninterrupted singlet oxygen generation to enhance breast cancer immunotherapy. Cell Rep. Phys. Sci..

[27] Bang C., Park M.G., Cho I.K., Lee D.E., Kim G.L., Jang E.H., Shim M.K., Yoon H.Y., Lee S., Kim J.H. (2023). Liposomes targeting the cancer cell-exposed receptor, claudin-4, for pancreatic cancer chemotherapy. Biomater. Res..

[28] Ning S., Suo M., Huang Q., Gao S., Qiao K., Lyu M., Huang Q., Zhang T., Tang B.Z. (2024). Biomimetic fusion liposomes boosting antitumor immunity and promote memory T cell differentiation to inhibit postoperative recurrence of breast cancer. Nano Today.

[29] Zhao Z., Wang W., Wang G., Huang Z., Zhou L., Lin L., Ou Y., Huang W., Zhang X., Wu C. (2023). Dual peptides-modified cationic liposomes for enhanced Lung cancer gene therapy by a gap junction regulating strategy. J. Nanobiotechnol..

[30] Alimu G., Yan T., Zhu L., Du Z., Ma R., Fan H., Chen S., Alifu N., Zhang X. (2023). Liposomes loaded with dual clinical photosensitizers for enhanced photodynamic therapy of cervical cancer. RSC Adv..

[31] Fulton M.D., Najahi-Missaoui W. (2023). Liposomes in Cancer Therapy: How Did We Start and Where Are We Now. Int. J. Mol. Sci..

[32] Fidan Y., Muçaj S., Timur S.S., Gürsoy R.N. (2024). Recent advances in liposome-based targeted cancer therapy. J. Liposome Res..

[33] Bustanji Y., Taneera J., Semreen M.H., Abu-Gharbieh E., El-Huneidi W., Faris M.A.I.E., Alzoubi K.H., Soares N.C., Albustanji B., Abuhelwa A.Y. (2023). Gold nanoparticles and breast cancer: A bibliometric analysis of the current state of research and future directions. OpenNano.

[34] Aria M., Cuccurullo C. (2017). bibliometrix: An R-tool for comprehensive science mapping analysis. J. Informetr..

[35] Bustanji Y., Shihab K.H.A., El-Huneidi W., Semreen M.H., Abu-Gharbieh E., Alzoubi K.H., Alqudah M.A.Y., Abuhelwa A.Y., Abu-Rish E.Y., Bajes H. (2023). Analysis and mapping of global scientific research on human monkeypox over the past 20 years. Vet. World.

[36] Moral-Munoz J., Herrera-Viedma E., Espejo A., Cobo M. (2020). Software tools for conducting bibliometric analysis in science: An up-to-date review. Prof. Inf..

[37] van Eck N.J., Waltman L. (2009). Software survey: VOSviewer, a computer program for bibliometric mapping. Proceedings of the 12th International Conference on Scientometrics and Informetrics.

[38] Wallin J.A. (2005). Bibliometric methods: Pitfalls and possibilities. Basic Clin. Pharmacol. Toxicol..

[39] Salmerón-Manzano E., Manzano-Agugliaro F. (2020). Bibliometric studies and worldwide research trends on global health. Int. J. Environ. Res. Public Health.

[40] Bustanji Y., Taneera J., Bargooth A., Abuhelwa A., Issa A., El-Huneidi W., Abu-Gharbieh E., H. Alzoubi K., Alqudah M.A.Y., Alhusban A. (2024). Exploring the global landscape of self-medication among students: Trends, risks, and recommendations for safe and responsible practices. Pharm. Pract..

[41] Van Eck N.J., Waltman L. (2010). Software survey: VOSviewer, a computer program for bibliometric mapping. Scientometrics.

[42] Olive K.P., Jacobetz M.A., Davidson C.J., Gopinathan A., McIntyre D., Honess D., Madhu B., Goldgraben M.A., Caldwell M.E., Allard D. (2009). Inhibition of Hedgehog signaling enhances delivery of chemotherapy in a mouse model of pancreatic cancer. Science.

[43] Cabral H., Matsumoto Y., Mizuno K., Chen Q., Murakami M., Kimura M., Terada Y., Kano M.R., Miyazono K., Uesaka M. (2011). Accumulation of sub-100 nm polymeric micelles in poorly permeable tumours depends on size. Nat. Nanotechnol..

[44] Kamerkar S., Lebleu V.S., Sugimoto H., Yang S., Ruivo C.F., Melo S.A., Lee J.J., Kalluri R. (2017). Exosomes facilitate therapeutic targeting of oncogenic KRAS in pancreatic cancer. Nature.

[45] Ding J., Wang K., Liu W., She Y., Sun Q., Shi J., Sun H., Wang D.C., Shao F. (2016). Pore-forming activity and structural autoinhibition of the gasdermin family. Nature.

[46] Arruebo M., Fernández-Pacheco R., Ibarra M.R., Santamaría J. (2007). Magnetic nanoparticles for drug delivery. Nano Today.

[47] O’Brien M.E.R., Wigler N., Inbar M., Rosso R., Grischke E., Santoro A., Catane R., Kieback D.G., Tomczak P., Ackland S.P. (2004). Reduced cardiotoxicity and comparable efficacy in a phase III trial of pegylated liposomal doxorubicin HCl (CAELYX™/Doxil^®^) versus conventional doxorubicin for first-line treatment of metastatic breast cancer. Ann. Oncol..

[48] Ito A., Shinkai M., Honda H., Kobayashi T. (2005). Medical application of functionalized magnetic nanoparticles. J. Biosci. Bioeng..

[49] van der Pol E., Böing A.N., Harrison P., Sturk A., Nieuwland R. (2012). Classification, functions, and clinical relevance of extracellular vesicles. Pharmacol. Rev..

[50] Alkilany A.M., Murphy C.J. (2010). Toxicity and cellular uptake of gold nanoparticles: What we have learned so far?. J. Nanopart. Res..

[51] Sun D., Zhuang X., Xiang X., Liu Y., Zhang S., Liu C., Barnes S., Grizzle W., Miller D., Zhang H.G. (2010). A novel nanoparticle drug delivery system: The anti-inflammatory activity of curcumin is enhanced when encapsulated in exosomes. Mol. Ther..

[52] Aiedeh K.M., Taha M.O., Al-Hiari Y., Bustanji Y., Alkhatib H.S. (2007). Effect of ionic crosslinking on the drug release properties of chitosan diacetate matrices. J. Pharm. Sci..

[53] Khdair A., Hamad I., Alkhatib H., Bustanji Y., Mohammad M., Tayem R., Aiedeh K. (2016). Modified-chitosan nanoparticles: Novel drug delivery systems improve oral bioavailability of doxorubicin. Eur. J. Pharm. Sci..

[54] Yeh P.Y., Chen J.Y., Shen M.Y., Che T.F., Lim S.C., Wang J., Tsai W.S., Frank C.W., Huang C.J., Chang Y.C. (2023). Liposome-tethered supported lipid bilayer platform for capture and release of heterogeneous populations of circulating tumor cells. J. Mater. Chem. B.

[55] Askarizadeh A., Mashreghi M., Mirhadi E., Mirzavi F., Shargh V.H., Badiee A., Alavizadeh S.H., Arabi L., Jaafari M.R. (2023). Doxorubicin-loaded liposomes surface engineered with the matrix metalloproteinase-2 cleavable polyethylene glycol conjugate for cancer therapy. Cancer Nanotechnol..

[56] Fu S., Chang L., Liu S., Gao T., Sang X., Zhang Z., Mu W., Liu X., Liang S., Yang H. (2023). Temperature sensitive liposome based cancer nanomedicine enables tumour lymph node immune microenvironment remodelling. Nat. Commun..

[57] Zou J. (2023). Site-specific delivery of cisplatin and paclitaxel mediated by liposomes: A promising approach in cancer chemotherapy. Environ. Res..

[58] AlKhatib H.S., Taha M.O., Aiedeh K.M., Bustanji Y., Sweileh B. (2006). Synthesis and in vitro behavior of iron-crosslinked N-methyl and N-benzyl hydroxamated derivatives of alginic acid as controlled release carriers. Eur. Polym. J..

[59] Cho E., Mun S.J., Jeon M., Kim H.K., Baek H., Ham Y.S., Gil W.J., Kim J.W., Yang C.S. (2023). Tumor-targeted liposomes with platycodin D2 promote apoptosis in colorectal cancer. Mater. Today Bio.

[60] Saraf S., Jain S.K. (2023). pH-sensitive liposomes bearing a chemotherapeutic agent and a natural apoptosis modulator for effective intracellular delivery to the solid tumor. Drug Deliv. Transl. Res..

[61] El-Hamid E.S.A., Gamal-Eldeen A.M., Sharaf Eldeen A.M. (2019). Liposome-coated nano doxorubicin induces apoptosis on oral squamous cell carcinoma CAL-27 cells. Arch. Oral Biol..

[62] Feuser P.E., De Pieri E., Oliveira M.E., Cordeiro A.P., Cercena R., Hermes de Araújo P.H., Dal Bó A.G., Machado-de-Ávila R.A. (2022). Cisplatin and paclitaxel-loaded liposomes induced cervical cancer (HeLa) cell death with multiple copies of human papillomavirus by apoptosis and decreased their cytotoxic effect on non-tumor cells. J. Drug Deliv. Sci. Technol..

[63] Wan S., Fan Q., Wu Y., Zhang J., Qiao G., Jiang N., Yang J., Liu Y., Li J., Chiampanichayakul S. (2023). Curcumin-Loaded Platelet Membrane Bioinspired Chitosan-Modified Liposome for Effective Cancer Therapy. Pharmaceutics.

[64] Zhang J., Huang Y., Xu J., Zhao R., Xiong C., Habu J., Wang Y., Luo X. (2022). Global publication trends and research hotspots of curcumin application in tumor: A 20-year bibliometric approach. Front. Oncol..

[65] Zhu Y., Wang A., Zhang S., Kim J., Xia J., Zhang F., Wang D., Wang Q., Wang J. (2023). Paclitaxel-loaded ginsenoside Rg3 liposomes for drug-resistant cancer therapy by dual targeting of the tumor microenvironment and cancer cells. J. Adv. Res..

[66] Li R., Liang H., Li J., Shao Z., Yang D., Bao J., Wang K., Xi W., Gao Z., Guo R. (2024). Paclitaxel liposome (Lipusu) based chemotherapy combined with immunotherapy for advanced non-small cell lung cancer: A multicenter, retrospective real-world study. BMC Cancer.

[67] Rak J., Kabesova M., Benes J., Pouckova P., Vetvicka D. (2023). Advances in Liposome-Encapsulated Phthalocyanines for Photodynamic Therapy. Life.

[68] Shi C., Li M., Zhang Z., Yao Q., Shao K., Xu F., Xu N., Li H., Fan J., Sun W. (2020). Catalase-based liposomal for reversing immunosuppressive tumor microenvironment and enhanced cancer chemo-photodynamic therapy. Biomaterials.

[69] Fahmy S.A., Azzazy H.M.E.S., Schaefer J. (2021). Liposome photosensitizer formulations for effective cancer photodynamic therapy. Pharmaceutics.

[70] Sesarman A., Tefas L., Sylvester B., Licarete E., Rauca V., Luput L., Patras L., Porav S., Banciu M., Porfire A. (2019). Co-delivery of curcumin and doxorubicin in PEGylated liposomes favored the antineoplastic C26 murine colon carcinoma microenvironment. Drug Deliv. Transl. Res..

[71] Feng X., Pi C., Fu S., Yang H., Zheng X., Hou Y., Wang Y., Zhang X., Zhao L., Wei Y. (2020). Combination of Curcumin and Paclitaxel Liposomes Exhibits Enhanced Cytotoxicity Towards A549/A549-T Cells and Unaltered Pharmacokinetics. J. Biomed. Nanotechnol..

[72] Alanazi A., Fadda L., Alhusaini A., Ahmad R. (2020). Antioxidant, antiapoptotic, and antifibrotic effects of the combination of liposomal resveratrol and carvedilol against doxorubicin-induced cardiomyopathy in rats. J. Biochem. Mol. Toxicol..

[73] Duan H., Liu C., Hou Y., Liu Y., Zhang Z., Zhao H., Xin X., Liu W., Zhang X., Chen L. (2022). Sequential Delivery of Quercetin and Paclitaxel for the Fibrotic Tumor Microenvironment Remodeling and Chemotherapy Potentiation via a Dual-Targeting Hybrid Micelle-in-Liposome System. ACS Appl. Mater. Interfaces.

[74] Zhang Q., Liu C., Wang J. (2019). Synergistic Effect of Quercetin and Vitamin C in Reducing Acute Toxicity and Improving Antitumor Activity of Liposomal Doxorubicin. Curr. Top. Nutraceutical Res..

[75] Mureşan M., Olteanu D., Filip G.A., Clichici S., Baldea I., Jurca T., Pallag A., Marian E., Frum A., Gligor F.G. (2021). Comparative study of the pharmacological properties and biological effects of polygonum aviculare l. Herba extract-entrapped liposomes versus quercetin-entrapped liposomes on doxorubicin-induced toxicity on huvecs. Pharmaceutics.

[76] Dorostkar H., Haghiralsadat B.F., Hemati M., Safari F., Hassanpour A., Naghib S.M., Roozbahani M.H., Mozafari M.R., Moradi A. (2023). Reduction of Doxorubicin-Induced Cardiotoxicity by Co-Administration of Smart Liposomal Doxorubicin and Free Quercetin: In Vitro and In Vivo Studies. Pharmaceutics.

[77] Ocaña-Arakachi K., Martínez-Herculano J., Jurado R., Llaguno-Munive M., Garcia-Lopez P. (2023). Pharmacokinetics and Anti-Tumor Efficacy of PEGylated Liposomes Co-Loaded with Cisplatin and Mifepristone. Pharmaceuticals.

[78] Tseu G.Y.W., Kamaruzaman K.A. (2023). A Review of Different Types of Liposomes and Their Advancements as a Form of Gene Therapy Treatment for Breast Cancer. Molecules.

[79] Alhamhoom Y., Kakinani G., Rahamathulla M., Ali M., Osmani R., Hani U., Yoonus Thajudeen K., Kiran Raj G., Gowda D.V. (2023). Recent advances in the liposomal nanovesicles based immunotherapy in the treatment of cancer: A review. Saudi Pharm. J..

[80] Chang R., Chu X., Zhang J., Fu R., Feng C., Jia D., Wang R., Yan H., Li G., Li J. (2023). Liposome-Based Co-Immunotherapy with TLR Agonist and CD47-SIRPα Checkpoint Blockade for Efficient Treatment of Colon Cancer. Molecules.

[81] Liu X., Yi X., Gu J., Ji Z., Zhu M., Shen M., Ren Y., Guo L., Liu T., Ding N. (2023). Immunoregulatory liposomes hitchhiking on neutrophils for enhanced carbon ion radiotherapy-assisted immunotherapy of glioblastoma. Nano Today.

[82] Lee E.K., Xiong N., Cheng S.C., Barry W.T., Penson R.T., Konstantinopoulos P.A., Hoffman M.A., Horowitz N., Dizon D.S., Stover E.H. (2020). Combined pembrolizumab and pegylated liposomal doxorubicin in platinum resistant ovarian cancer: A phase 2 clinical trial. Gynecol. Oncol..

[83] Ma G.L., Lin W.F. (2023). Immune checkpoint inhibition mediated with liposomal nanomedicine for cancer therapy. Mil. Med. Res..

[84] Huang Z., Wei G., Zeng Z., Huang Y., Huang L., Shen Y., Sun X., Xu C., Zhao C. (2019). Enhanced cancer therapy through synergetic photodynamic/immune checkpoint blockade mediated by a liposomal conjugate comprised of porphyrin and IDO inhibitor. Theranostics.

[85] Moghassemi S., Dadashzadeh A., Azevedo R.B., Feron O., Amorim C.A. (2021). Photodynamic cancer therapy using liposomes as an advanced vesicular photosensitizer delivery system. J. Control. Release.

[86] Ghosh S., Carter K.A., Lovell J.F. (2019). Liposomal formulations of photosensitizers. Biomaterials.

[87] Xiao Z., Zhuang B., Zhang G., Li M., Jin Y. (2021). Pulmonary delivery of cationic liposomal hydroxycamptothecin and 5-aminolevulinic acid for chemo-sonodynamic therapy of metastatic lung cancer. Int. J. Pharm..

[88] Peng P.C., Hong R.L., Tsai T., Chen C.T. (2019). Co-encapsulation of chlorin e6 and chemotherapeutic drugs in a pegylated liposome enhance the efficacy of tumor treatment: Pharmacokinetics and therapeutic efficacy. Pharmaceutics.

[89] Zhu Y.X., Jia H.R., Duan Q.Y., Liu X., Yang J., Liu Y., Wu F.G. (2020). Photosensitizer-Doped and Plasma Membrane-Responsive Liposomes for Nuclear Drug Delivery and Multidrug Resistance Reversal. ACS Appl. Mater. Interfaces.

[90] Nsairat H., Mahmoud I.S., Odeh F., Abuarqoub D., Al-Azzawi H., Zaza R., Qadri M.I., Ismail S., Al Bawab A., Awidi A. (2020). Grafting of anti-nucleolin aptamer into preformed and remotely loaded liposomes through aptamer-cholesterol post-insertion. RSC Adv..

[91] Honari A., Merillat D.A., Bellary A., Ghaderi M., Sirsi S.R. (2021). Improving release of liposome-encapsulated drugs with focused ultrasound and vaporizable droplet-liposome nanoclusters. Pharmaceutics.

[92] Yazdian-Robati R., Amiri E., Kamali H., Khosravi A., Taghdisi S.M., Jaafari M.R., Mashreghi M., Moosavian S.A. (2023). CD44-specific short peptide A6 boosts cellular uptake and anticancer efficacy of PEGylated liposomal doxorubicin in vitro and in vivo. Cancer Nanotechnol..

[93] Silli E.K., Li M., Shao Y., Zhang Y., Hou G., Du J., Liang J., Wang Y. (2023). Liposomal nanostructures for Gemcitabine and Paclitaxel delivery in pancreatic cancer. Eur. J. Pharm. Biopharm..

[94] Raza F., Evans L., Motallebi M., Zafar H., Pereira-Silva M., Saleem K., Peixoto D., Rahdar A., Sharifi E., Veiga F. (2023). Liposome-based diagnostic and therapeutic applications for pancreatic cancer. Acta Biomater..

[95] Aloss K., Hamar P. (2023). Recent Preclinical and Clinical Progress in Liposomal Doxorubicin. Pharmaceutics.

[96] Mirzavi F., Barati M., Soleimani A., Vakili-Ghartavol R., Jaafari M.R., Soukhtanloo M. (2021). A review on liposome-based therapeutic approaches against malignant melanoma. Int. J. Pharm..

[97] Alavi M., Varma R.S. (2020). Overview of novel strategies for the delivery of anthracyclines to cancer cells by liposomal and polymeric nanoformulations. Int. J. Biol. Macromol..

[98] Nsairat H., Alshaer W., Odeh F., Esawi E., Khater D., Bawab A.A., El-Tanani M., Awidi A., Mubarak M.S. (2023). Recent advances in using liposomes for delivery of nucleic acid-based therapeutics. OpenNano.

[99] Sharma M., Sudha Ambadipudi S.S.S.S., Kumar Chouhan N., Lakshma Nayak V., Pabbaraja S., Balaji Andugulapati S., Sistla R. (2023). Design, synthesis and biological evaluation of novel cationic liposomes loaded with melphalan for the treatment of cancer. Bioorg. Med. Chem. Lett..

[100] Li M., Li S., Li Y., Li X., Yang G., Li M., Xie Y., Su W., Wu J., Jia L. (2022). Cationic liposomes co-deliver chemotherapeutics and siRNA for the treatment of breast cancer. Eur. J. Med. Chem..

[101] Abu Lila A.S., Ishida T., Kiwada H. (2010). Targeting anticancer drugs to tumor vasculature using cationic liposomes. Pharm. Res..

[102] Majzoub R.N., Ewert K.K., Safinya C.R. (2016). Cationic liposome-nucleic acid nanoparticle assemblies with applications in gene delivery and gene silencing. Philos. Trans. R. Soc. A Math. Phys. Eng. Sci..

[103] Gladkikh D.V., Sen′ Kova A.V., Chernikov I.V., Kabilova T.O., Popova N.A., Nikolin V.P., Shmendel E.V., Maslov M.A., Vlassov V.V., Zenkova M.A. (2021). Folate-equipped cationic liposomes deliver anti-mdr1-sirna to the tumor and increase the efficiency of chemotherapy. Pharmaceutics.

[104] Sousa D.A., Gaspar R., Ferreira C.J.O., Baltazar F., Rodrigues L.R., Silva B.F.B. (2022). In Vitro CRISPR/Cas9 Transfection and Gene-Editing Mediated by Multivalent Cationic Liposome–DNA Complexes. Pharmaceutics.

[105] Zhang J., Guan M., Ma C., Liu Y., Lv M., Zhang Z., Gao H., Zhang K. (2023). Highly Effective Detection of Exosomal miRNAs in Plasma Using Liposome-Mediated Transfection CRISPR/Cas13a. ACS Sens..

[106] Desai D., Gaud R.S., Shende P. (2021). Potential of Chimeric Antigen Receptor T-Cells in Cancer Therapy. Advances in Experimental Medicine and Biology.

[107] Huang C.H., Dong T., Phung A.T., Shah J.R., Larson C., Sanchez A.B., Blair S.L., Oronsky B., Trogler W.C., Reid T. (2022). Full Remission of CAR-Deficient Tumors by DOTAP-Folate Liposome Encapsulation of Adenovirus. ACS Biomater. Sci. Eng..

[108] Ding Y., Wang L., Li H., Miao F., Zhang Z., Hu C., Yu W., Tang Q., Shao G. (2022). Application of lipid nanovesicle drug delivery system in cancer immunotherapy. J. Nanobiotechnol..

